# Nanoparticles of the Perovskite-Structure CaTiO_3_ System: The Synthesis, Characterization, and Evaluation of Its Photocatalytic Capacity to Degrade Emerging Pollutants

**DOI:** 10.3390/nano13222967

**Published:** 2023-11-17

**Authors:** Lizet Cerón-Urbano, Carol J. Aguilar, Jesús E. Diosa, Edgar Mosquera-Vargas

**Affiliations:** 1 Grupo de Transiciones de Fase y Materiales Funcionales, Departamento de Física, Facultad de Ciencias Naturales y Exactas, Universidad del Valle, Santiago de Cali 760042, Colombia; neida.ceron@correounivalle.edu.co (L.C.-U.); carol.aguilar@correounivalle.edu.co (C.J.A.); jesus.diosa@correounivalle.edu.co (J.E.D.); 2 Centro de Excelencia en Nuevos Materiales (CENM), Universidad del Valle, Santiago de Cali 760042, Colombia

**Keywords:** emerging pollutants, polymer precursor method, optical properties, photocatalysis, degradation, water treatment, methyl orange, levofloxacin

## Abstract

In this research work, the photocatalytic capacity shown by the nanoparticles of the CaTiO_3_ system was evaluated to degrade two pollutants of emerging concern, namely methyl orange (MO)—considered an organic contaminating substance of the textile industry that is non-biodegradable when dissolved in water—and levofloxacin (LVF), an antibiotic widely used in the treatment of infectious diseases that is released mostly to the environment in its original chemical form. The synthesis process used to obtain these powders was the polymeric precursor method (Pechini), at a temperature of 700 °C for 6 h. The characterization of the obtained oxide nanoparticles of interest revealed the presence of a majority perovskite-type phase with an orthorhombic Pbnm structure and a minority rutile-type $TiO_{2}$ phase, with a P42/mnm structure and a primary particle size <100nm. The adsorption–desorption isotherms of the synthesized solids had H3-type hysteresis loops, characteristic of mesoporous solids, with a BET surface area of $10.01\;m^{2}/g$. The Raman and FTIR spectroscopy results made it possible to identify the characteristic vibrations of the synthesized system and the characteristic deformations of the perovskite structure, reiterating the results obtained from the XRD analysis. Furthermore, a bandgap energy of ~3.4eV and characteristic emissions in the violet (437 nm/2.8 eV) and orange (611 nm/2.03 eV) were determined for excitation lengths of 250 nm and 325 nm, respectively, showing that these systems have a strong emission in the visible light region and allowing their use in photocatalytic activity to be potentialized. The powders obtained were studied for their photocatalytic capacity to degrade methyl orange (MO) and levofloxacin (LVF), dissolved in water. To quantify the coloring concentration, UV–visible spectroscopy was used considering the variation in the intensity of the characteristic of the greatest absorption, which correlated with the change in the concentration of the contaminant in the solution. The results showed that after irradiation with ultraviolet light, the degradation of the contaminants MO and LVF was 79.4% and 98.1% with concentrations of 5 g/L and 10 g/L, respectively.

## 1. Introduction

Emerging pollutants (EPs) are synthetic or naturally occurring chemicals or biological pollutants whose presence in the environment is not considered significant in terms of distribution and concentration, so they tend not to be monitored [1,2]. There is currently a growing interest in emerging pollutants, since they cause environmental problems and risks to health. These compounds are disseminated in the environment and have been detected in water supply sources, groundwater, and even in drinking water. They are compounds of which relatively little is known, in terms of their presence, impact, and treatment. In most cases, they are unregulated contaminants, which may be candidates for future regulation, depending on research on their potential health effects and monitoring data regarding their incidence in the environment; therefore, they are very much worth researching [1]. Among the main EPs are pharmaceuticals, personal and animal care products, pesticides, flame retardants, industrial additives, food additives, lifestyle products, illicit drugs, perfluorinated compounds, hormones, microplastics, engineered nanomaterials, resistance genes, and dyes [1,3,4,5,6,7]. Nevertheless, the list is long and not definitive, because more substances emerge every day that can be included. Table 1 presents a division that is common to several studies [4].

The main sources of EPs are domestic wastewater, hospital effluents, industrial wastewater, agricultural, livestock and aquaculture runoff, and landfill leachate, with the effluent from wastewater treatment plants being the main contributor to the occurrence of EPs in bodies of water [8]. This is mainly because wastewater treatment plants are not designed to remove this type of pollution. The discharged effluent thus contains organic substances and metabolites which may reach the environment worldwide [8]. Even when usually detected at low concentrations, from µgL^−1^ to ngL^−1^ and rarely in mgL^−1^, EPs are capable of causing adverse effects in organisms [9]. Their effects range from alterations in the immune system of aquatic animals to mutations and cancer in humans [4,8]. Perfluorinated compounds, personal care products, engineered nanomaterials, antibiotics, and resistance genes are considered as endocrine disruptors [7]. Despite their presence in such low quantities, they have shown various levels of ecotoxicity, cytotoxicity, and genotoxicity in the environment. Several studies have been conducted on the toxicity of emerging contaminants, and they have been found to cause both acute and chronic effects in different organisms ranging from aquatic organisms to human beings (see Table 2) [10].

The presence of EPs is increasing day by day due to the inability of conventional treatments to remove them. In addition, they can accumulate in the trophic chain and migrate to groundwater sources, or be deposited in sediments [4]. Normal organic matter can be degraded easily through water purification. Eps, on the other hand, can barely be removed this way due to five features: serious harm, hidden risks, environmental persistence, wide sources, and complex governance. The efficient removal of EPs in water has thus become one of the most pressing topics for researchers [2]. Pharmaceuticals and dyes are among the contaminants that are increasingly being identified in bodies of water. They are resistant and difficult to remove in conventional wastewater treatment plants [6].

Global health problems have contributed to the expansion of the pharmaceutical industries and the consumption of medicines. The incorrect disposal of these compounds and the excretion by animals and humans of up to 90% of the administered drugs represent a threat to various environments, since their active principles are constantly found in small amounts in drinking water, wastewater, and surface water, interacting with biological systems, poisoning animals, and harming human health [13,14]. Drugs from the therapeutic classes of analgesics/nonsteroidal anti-inflammatory drugs, antibiotics, beta-blockers, and psychiatric drugs have been extensively detected worldwide at moderately high concentrations (up to hundreds of µg) [15]. Since the COVID-19 outbreak, approximately 70% of hospitalized patients have been treated with antibiotics. Levofloxacin (LVF) is an antibiotic that is widely used in the treatment of infectious diseases [16], and due to its broad-spectrum antibacterial activity, it is also used in animal husbandry and agriculture [17,18]. However, only a small part of LVF is metabolized or absorbed in the body, and most is released into the environment in its original form [16], generally due to the high stability of LVF molecules, most of which will be metabolized in the natural environment in the form of active species after being consumed, causing irreversible damage to the ecological environment [19] due to their persistence towards biodegradation [20].

Additionally, around the world, about $7\times 10^{5}$ tons of inks are produced annually. Wastewater that is contaminated with dyes from the textile, paper, pharmaceutical, and other industries is also discharged into the environment in large quantities [16]. Dyes have thus become one of the main sources of water pollution, and methyl orange (MO) is one of the most widely used organic dyes in the textile industry [21]. The discharge of large amounts of antibiotics and organic dyes causes water pollution and seriously threatens aquatic organisms and human society. Therefore, it is crucial to search for a green and efficient treatment technology for the degradation of LVF and MO [16].

Technologies of a physicochemical nature have been designed, such as membrane filtration or adsorption using activated carbon, which are expensive and commercially unattractive. Furthermore, these processes are limited to transferring contaminants from one phase to another instead of removing them [22]. Various technologies have been explored for the treatment of water contaminated with antibiotics, among which the following stand out: chemical oxidation, physical adsorption, and biodegradation [23]. Among the conventional technologies used for the treatment of waters that contain dyes are ion exchange [24], precipitation [22], chlorination [25], reverse osmosis [26], flocculation, and coagulation [27,28,29]. Such conventional treatments, used for the decontamination of water, are procedures that are inadequate to reach the degree of purity required by law or to enable the subsequent use of the treated effluent [30].

This has led to considering, as a good alternative, what are known as advanced oxidation technologies or processes (AOTs or AOPs, respectively) that are not as widely applied as they ought to be and, worse still, are not widely disseminated in emerging economies like those of Latin America. Most AOTs can be applied to the remediation and removal of contaminants from special waters, generally on a small or medium scale [30]. The methods can be used alone or in combination, among themselves or with conventional methods, and can also be applied to contaminants in air and soil, even allowing disinfection through the inactivation of bacteria and viruses [30]. AOPs are based on physicochemical processes capable of producing profound changes in the chemical structure of pollutants. They are radical-mediated oxidation processes that have the capacity for the complete mineralization of organic compounds [31]. 

The concept was initially established by Glaze et al. [30], who defined AOPs as processes that involve the generation and use of highly oxidizing transient species, such as the hydroxyl radical (•OH), which can be generated mainly through photochemical means (including sunlight) or using other forms of energy, and which has a high capacity to oxidize organic matter [30]. Among them, photocatalytic technology is considered the most promising method because it is economical, reusable, and ecological [16]. Photocatalysis has been widely reported for the degradation of dyes and antibiotics; several investigations have recently been published on the degradation of dyes [31,32,33,34,35,36] and on the degradation of antibiotics, specifically LVF [16,17,18,19,20]. 

Some inorganic semiconductor photocatalysts such as $TiO_{2}$, $CaTiO_{3},$ and CdS have been developed and applied in photocatalysis. When the photocatalyst absorbs light energy, electrons $\left(e^{-}\right)$ and holes $\left(h^{+}\right)$ are generated on the surface of the material. The $e^{-}$ and $h^{+}$ can induce the redox reaction of $O_{2}$ and $H_{2}O$ to generate a superoxide radical $\left(•O_{2}^{-}\right)$ and a hydroxyl radical (•OH), which have a strong potential for the direct oxidation of organic contaminants to $CO_{2}$ and $H_{2}O$ or small molecule species. 

However, despite great efforts in the development of efficient photocatalysts, traditional photocatalysts absorb only ultraviolet light and offer lower quantum efficiency, which limits their implementation on an industrial scale. Therefore, to overcome these limitations, the development of stable and efficient visible light photocatalysts is necessary. The design of new organic semiconductor photocatalysts offers an alternative in improving the surface area, adjusting the internal pore size and high porosity, providing additional reaction sites, which increases the photoreactivity of these materials. Photocatalytic water splitting using polymeric semiconductors to produce hydrogen has promising applications but is still in the fundamental stage at the laboratory scale [37]. Some research on polymeric materials has shown their photocatalytic efficiency in the degradation of contaminants; examples of these materials are coordination polymers (CPs) [38] and metal–organic structures (MOFs) [39]. The exploration of appropriate building blocks remains a challenge for the development of highly efficient polymeric photocatalysts, and an alternative in future work may be to expand procedures to combine photocatalysis (with organic, transition metal, and inorganic semiconductors), green chemistry, and nanotechnology [40]. 

Elsewhere, perovskite-type catalytic materials for heterogeneous photocatalysis are mixed metal oxides that are gaining relevance due to their structural flexibility [40], increase in surface area [41], high extinction coefficients, optimal band gaps, high photoluminescent quantum yields, and long electron–hole diffusion lengths [42]. Among numerous photocatalysts, $CaTiO_{3}$ (CTO), as a typical perovskite-type metal oxide, has shown potential application prospects in the field of photocatalysis due to its high stability and excellent photoelectric catalytic activity [16]. However, despite the important contributions of research work referring to the synthesis processes of structures of the $CaTiO_{3}$ system, the study of the synthesis conditions and their influence on the structural, optical, and physicochemical properties in these materials continue as research topics, mainly given their potential uses in the degradation of contaminants from textile wastewater [43]. 

Among the most explored synthesis methods to obtain CTO are hydrothermal, solvothermal, co-precipitation, chemical vapor deposition, microwave [44], solid-state reaction [45], combustion, polyacrylamide gel, microwave-assisted hydrothermal, sol–gel, and electrospinning [46]. However, the control over the purity of the material is not clear in the different synthesis processes presented, given that some parameters, such as the sintering temperature, generate small percentages of secondary phases that can modify its catalytic capacity [47]. The different synthesis routes indicate that obtaining CaTiO_3_ powders and their possible applications in photocatalysis depend on the methodology, precursors used, concentrations, and thermal treatment temperature. Currently, alternative methods are being sought such as reactive spark plasma sintering (SPS) and spark plasma sintering–reaction synthesis (SPS-RS), which use high-speed heating (250–300 °C/min) of nanopowders up to the sintering temperature, using short electrical pulses with moderate pressure (<100 MPa). These methods have been used to obtain systems with a perovskite structure, being promising in improving the physical–chemical characteristics, since the formation of grain boundaries and surface diffusion is favored, which can improve the catalytic properties of these materials [41,48]. Another alternative process is the polymer precursor method, which has been little used in the synthesis of perovskite structures, despite having multiple advantages in obtaining mixed valence solids. The use of this method improves the problems of segregation or precipitation in the solution of the precursors, generating control in the purity of the material, mainly due to the fixation of the cations to the resin, thus allowing a better stoichiometry of the compound that it is sought to obtain. This research proposes to synthesize $CaTiO_{3}$ powders using the modified polymeric precursor method, which has not been reported in CTO synthesis processes. 

The study objective is to present the process of the synthesis and characterization of the perovskite-type $CaTiO_{3}$ ceramic powder and its capacity for the photodegradation of emerging contaminants, MO and LVF, driven by ultraviolet light. 

## 2. Materials and Methods

### 2.1. Preparation of $CaTiO_{3}$ Nanoparticles using the Pechini Polymeric Precursor Method

Ethylene glycol ($HOCH_{2}CH_{2}OH$, purity 99.8% - Merck Millipore) was heated to a temperature of 70 °C, then citric acid (C_6_H_8_O_7_⋅H_2_O, purity 99.5%—Merck Millipore) was added in the solid phase whilst maintaining a steady temperature and stirring constantly until a clear solution was obtained. Once the mixture was homogeneous, it was brought to room temperature and titanium tetrabutoxide ($Ti[O{\left(CH_{2}\right)}_{3}CH_{3}$]_4_, purity of 97.0%—Sigma Aldrich), and calcium acetate ($Ca{\left(CH_{3}COO\right)}_{2}$, purity 94% - Sigma Aldrich) were slowly added.

The uniform mixture of ethylene glycol, citric acid, and the precursors of the cations of interest was brought to a suitable pH by slowly adding ammonium hydroxide ($NH_{3}$ in $H_{2}O$ at 28%, purity ≥99.99% - Merck Millipore). Process control was carried out with the Hanna Instruments 2021 pH-meter, working with a glass electrode previously calibrated with the buffer solutions of pH 4, pH 7, and pH 10. The solution was subsequently heated to a moderate temperature of ~130 °C, with continuous stirring, to promote the esterification and polyesterification processes, until a transparent polymeric resin was formed. This resin was calcined between 200 and 300 °C to remove the solvent present in the system (pre-calcination process). The solid obtained from the pre-calcination was thermally treated at 700 °C, for 6 h, to remove the organic material and obtain the mixture of oxides of interest.

### 2.2. Potentiometric Titration Curve

The potentiometric titration curve of the solutions of the $CaTiO_{3}$ system was obtained by adding 0.5 mL of an ammonium hydroxide solution ($NH_{4}OH$ at 28%, purity ≥99.99% - Merck Millipore) with an Airco 160801-023 dispenser. The pH value of the systems, as well as the volume of the added base, was recorded periodically during the addition of $NH_{4}OH$ until reaching the stabilization pH value.

### 2.3. Structural and Optical Characterization of Ceramic Powders

In order to determine the most appropriate heat treatment temperatures in the synthesis process, evaluate the effect of heat treatment, and consider the different physicochemical events that might occur in the pre-calcined samples of the $CaTiO_{3}$ systems, differential scanning calorimetry (DSC) and thermogravimetric analysis (TGA) curves were obtained in a temperature range of 28 to 1000 °C, using a simultaneous thermal analyzer SDT Q600 V20.9 Build 20 from TA instruments, with an alumina cell, a nominal air flow of 100 mL/min, and a heating ramp of 10 °C/min. The crystalline phases present in the synthesized ceramic powders were identified by obtaining diffractograms of the $CaTiO_{3}$ system. The FEI Inspect S 50 scanning electron microscope was used to determine the size, shape, and state of agglomeration of the particles. The equipment used was the PANalytical X’Pert PRO MRD Diffractometer with a Cu source (λ = 1.54 Å), belonging to the X-ray diffraction laboratory of the Universidad del Valle, Santiago de Cali - Colombia. The tests were carried out at a voltage of 40kV at 2°/min, at a current of 30mA, in the measurement range of 20–100°. 

The results obtained were processed and analyzed using the X’pert HighScore 3.0.0 and GSAS 1.0.0 programs. The specific surface area, the volume, and the pore size distribution of the synthesized porous material were obtained via adsorption with $N_{2}$ and the BET method, using the 3 FLEX Micromeritics equipment. All samples were degassed at 200 °C before determining the adsorption isotherms. The functional groups present in the different samples of the systems obtained during the synthesis process were determined via FTIR spectroscopy. The Jasco FTIR 6800 spectrophotometer was used to carry out the test. The FTIR spectra obtained were performed at a resolution of $16\;{cm}^{-1}/s$. For detailed analysis of the previously obtained FTIR spectra, these were analyzed by using the Fityk 0.8.6 software at $800-400\;{cm}^{-1}$ using symmetrical Gaussian curves. 

To perform the Raman spectroscopy analysis, a JASCO Raman analyzer, model NRS-4500, was used, using a laser excitation source with a wavelength of 532nm and an integration time of 20 s, for each spectrum, in a range between 200 and $850\;{cm}^{-1}$. UV-Vis diffuse reflectance spectroscopy (UV-Vis-DRS) was used to determine the electronic transitions of solid structures when irradiated with ultraviolet or visible light. With the resulting absorption spectrum, it is possible to obtain the energy value of the bandwidth. The spectra of the synthesized powders were obtained with a JASCO V-700 UV-Vis-NIR spectrophotometer in the range between 190 and 800nm, and at a scanning speed of 100nm/min. A photoluminescence (PL) study of the synthesized samples was carried out in order to obtain information on the efficiency of the charge capture, migration, and transfer processes, as well as on the surface states and the presence of defects at room temperature (RT) using a JASCO FP-8250 spectrophotometer, with a xenon discharge lamp (150 W power) as the excitation source. 

### 2.4. Photocatalytic Evaluation of $CaTiO_{3}$ Nanoparticles 

To evaluate the degradation capacity of MO and LVF by the $CaTiO_{3}$ compounds, the UV-Vis spectroscopy technique was used. Initially, the relationship between the absorbance recorded with the spectrophotometer and the concentration of pollutants in the solution were determined, obtaining the contaminant calibration curve. UV-Vis absorption spectra were obtained using a JASCO V-700 spectrophotometer in the range of 190−700nm. Subsequently, solutions of MO (Sigma-Aldrich) and commercial LVF (Laproff laboratories, Colombia) with initial concentrations of 10 ppm were introduced into a photodegradation chamber with magnetic stirring at 300rpm and equipped with an 800 Watt Philips mercury lamp. The pH of the solution was set at 6.2 and 4.5 for the MO and LVF, respectively; the temperature of the systems was maintained at 37 °C. It was decided to study the kinetics of photodegradation using 3, 5, 10, and 15 g of catalyst for each L of MO and LVF at 10 ppm, under constant stirring and in times that varied between 15 and 180 min.

Initially, the nanoparticles were added to the solution with the contaminant. This mixture was located in the photoreactor in the absence of UV radiation and with magnetic stirring for 30 min in order to ensure the adsorption–desorption equilibrium on the catalyst surface. Once this period was completed, the reaction was started by turning on the lamp. To carry out the experiment, aliquots of the solution (~3 mL) were taken every 15 min, removing the nanoparticles by means of centrifugation at 7000× *g* rpm for two minutes. The degradation of the contaminants was analyzed through the decrease in intensity of the maximum absorption peak, located approximately at 464nm for MO and 293 nm for LVF.

From the spectra obtained, the absorbance values corresponding to the peaks of the maximum absorbance characteristics of MO and LVF were recorded. Using the calibration curve, the MO and LVF concentration values were obtained for each aliquot. With these concentration values, for the different times and the initial concentration of pollutants in the system, the normalized concentration curves were obtained. In addition, the percentage of removal of the contaminant from the solutions was calculated using the following expression:

(1) $$\%D=\frac{C_{o}-C_{f}}{C_{o}}\;*100$$

where $C_{o}$ and $C_{f}$ are the initial concentration values of the pollutant and the concentration at time “t”, respectively.

## 3. Results and Discussions

### 3.1. Potentiometric Titration Curves

**Figure 1:**
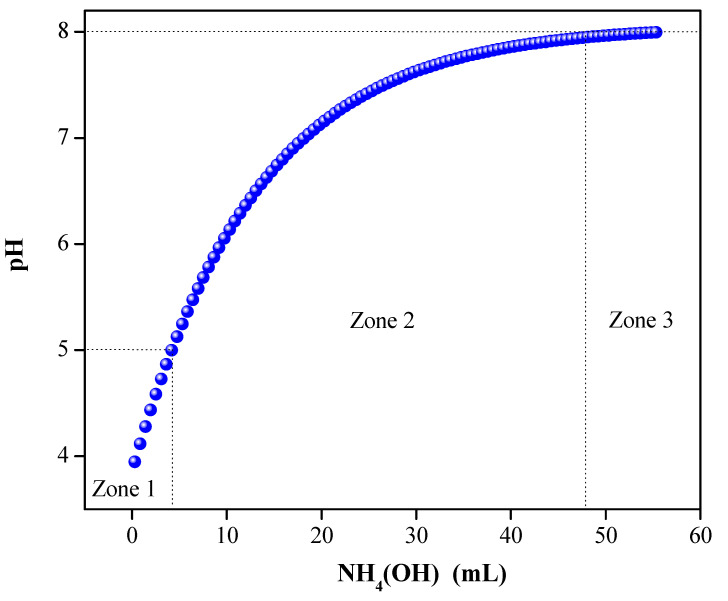
Potentiometric titration curve of the $CaTiO_{3}$ system.

To ensure the reproducibility of the method and the suitable characteristics of the final product, the potentiometric titration curves were made (Figure 1), which allow for the monitoring of the synthesis process, recording the variation in a physicochemical parameter (pH=−log[H+]) depending on the amount of $NH_{4}OH$ added to the system. The initial system, the solution of the precursor, had an opaque white color with a pH value between 4 and 5, such that, by adding ammonium hydroxide at a rate of 0.300mL/s, its pH increased, as illustrated in Figure 1, zone (I). In zone (II) of the process, when the pH was between 5 and 8, the system changed color, turning yellow. Finally, in zone (III), the pH stabilized until reaching a value between 8 and 8.2; in this region, the system gave an opaque yellow color.

### 3.2. Characterization of the Solid Phase

#### 3.2.1. Thermogravimetric Analysis (TGA) and Differential Scanning Calorimetry (DSC)

**Figure 2:**
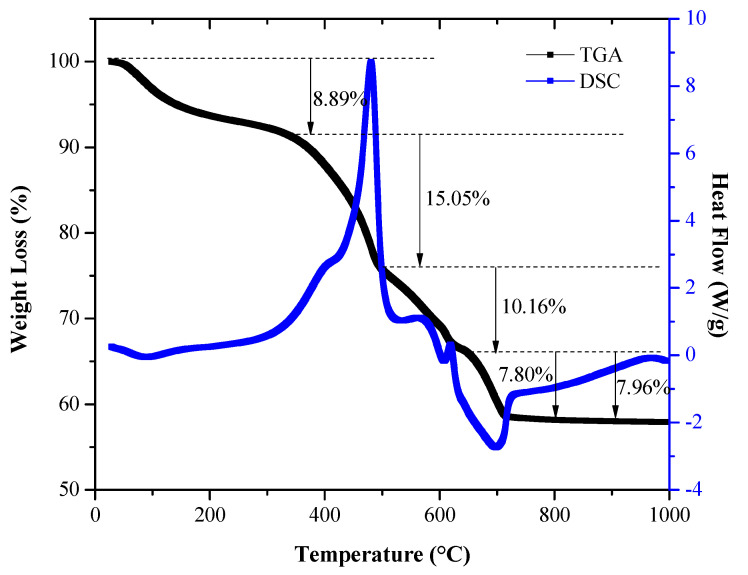
Thermal analysis corresponding to the pre-calcination that was used to obtain the $CaTiO_{3}$ system.

To establish the effect of heat treatment on the samples of the $CaTiO_{3}$ system and determine the optimal temperature for synthesis of the oxide of interest, TGA and DSC analyses were performed. Figure 2 shows the thermal evolution of the pre-calcined system obtained by using the Pechini method. The thermogravimetric curve (TGA Figure 2, black line) shows that four important physicochemical events occurred that produced mass losses in the system. These events were related to different thermal peaks that were observed in the calorimetry curve (DSC Figure 2, blue line). Thus, at a temperature of 90 °C, an endothermic peak occurred, associated with a weight loss of 6.31% up to 200 °C; this physicochemical event is related to the removal of water adsorbed on the surface of the powdered material [49,50]. Between 400 and 750 °C, three exothermic peaks were observed at ~481, 564, and 619 °C, which could be attributed to the oxidation of the organic phase present in the system, initially favoring the formation of oxy-carbonates and finally that of the oxide [49,50,51]. These exothermic peaks are assigned to mass losses of ~15, 10, and 7.8%, respectively (Figure 2). For temperatures higher than 700 °C, the weight loss of the sample was not appreciable. 

According to the results obtained, it can be observed that the most important physicochemical processes, which lead to obtaining the oxide of interest, occur below 700 °C. Above this temperature, the weight of the sample remains almost constant, which is a sign of the completion of the reactions and the formation of the crystalline phase of the compound [51]. This led to defining this temperature to thermally treat the pre-calcinate and obtain the $CaTiO_{3}$ of interest.

#### 3.2.2. Scanning Electron Microscope

**Figure 3:**
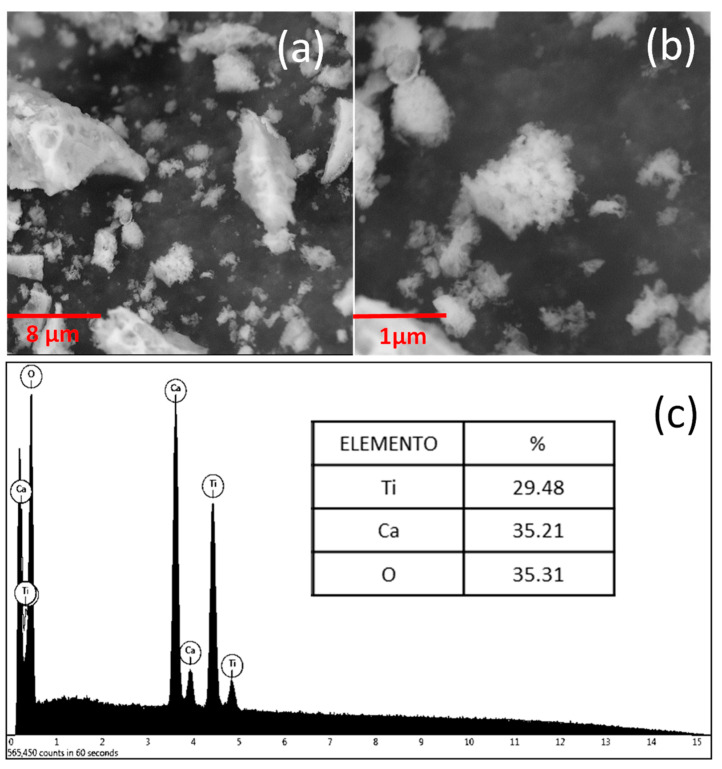
SEM Micrographs: (**a**) ×8700, (**b**) ×22,500, and (**c**) EDS.

Figure 3 shows the SEM micrographs for the powders of the $CaTiO_{3}$ systems. The micrographs show the agglomeration of the primary particles (Figure 3a), whose secondary particles have a random shape, made up of groups of disordered primary particles (Figure 3b). The size of the secondary particles (agglomerates) for these two compositions is close to 500 nm. Figure 3c shows the results of the elemental chemical analysis, performed with the EDS microprobe, in the region indicated by the box. When observing the EDS spectra of the sample, the Ca, Ti, and O were evidenced as the only elements present. Moreover, taking as a reference the theoretical percentages of each element, and considering the stoichiometry of the compound, when comparing these data with those obtained from the EDS analysis, an agreement with the calculated theoretical percentage is evident.

#### 3.2.3. X-ray Diffraction

**Figure 4:**
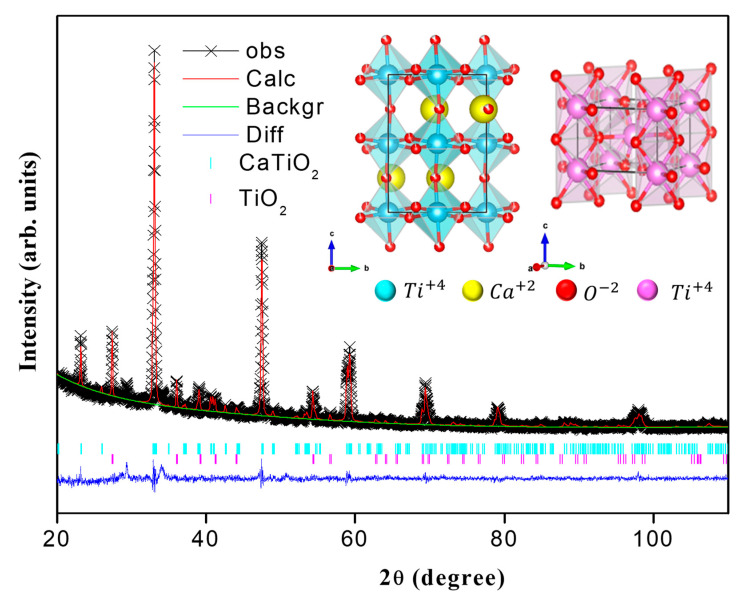
Results of the XRD diffractogram fit, using Rietveld refinement of the $CaTiO_{3}$ system heat-treated at 900 °C. The inset shows the structure of the solid seen from plane ab. (The yellow sphere represents the Ca atoms; the blue, the titanium atom; and the red, the oxygen atom).

Figure 4 shows the XRD patterns of the $CaTiO_{3}$ system samples synthesized at 700 °C. The analysis of the X-ray diffraction patterns (XRD) was carried out using the X’pert HighScore program and using the Rietveld refinement technique with the GSAS program. The Rietveld refinement results indicated that the sample presents a majority perovskite-type phase with a Pbnm orthorhombic structure and space group 62, and a minority phase of $TiO_{2}$, which was rutile-type with a P42/mnm structure and space group 136. The diffractogram shows sharp peaks, indicating the crystalline nature of the system, similar to the standard patterns corresponding to systems obtained from the same material [52,53,54].

The structural parameters obtained are recorded in Table 1. The average particle diameter was calculated from some parameters refined using the Rietveld method through the GSAS 1.0.0 software, considering a spherical morphology for the particles (k=0.94) and the wavelength of the CuKa radiation $\left(\lambda =1.5418\;Å\right),$ and averaging the perpendicular and parallel mean crystallite size. The average particle size for all the samples was close to 80 nm. The inset of the diffractogram in Figure 4 shows the structural model that was obtained using the Vesta 3.4.6 software, projected from the [002] plane. These structural models show the effect of the twist distortion of the TiO_6_ octahedral lattice, whose tolerance factor was recorded at 0.902. Calculation of the tolerance factor, for the sample synthesized in this work, was carried out using Equation (2).

(2) $$t=\frac{r_{Ca}+r_{o}}{\sqrt{2}\left(r_{Ti}+r_{o}\right)}$$

where $r_{Ca}$, $r_{Ti}$, and $r_{o}$ are the ionic radii of calcium, titanium, and oxygen, respectively. Typically, for values of t~1, the perovskite structure tends to have cubic symmetry with relatively right Ti−O−Ti bond angles. Low values of the tolerance factor suggest a high degree of internal deformation due to the difference in size, leading to distorted perovskite structures [55]. To determine the tolerance factor, the ionic radii were used—1.97 Å for Ca (cation A), 1.47 Å for Ti (cation B), and 0.74 Å for O^2−^ (anion) —and the results indicate that the factor is found in the interval of 0.75<t<0.91 (see Table 3), for which it can be affirmed that the samples correspond to stable perovskites, with orthorhombic symmetry. This is due to the difference in values between the ionic radii of the Ca and Ti cations, which would propitiate the inclination and rotation of the $TiO_{6}$ octahedrons.

It is important to consider the effect of the structural distortions of these systems on their different properties, which can be of two types, with the first related to the cooperative inclination of the $TiO_{6}$ octahedrons that is due to the effect of the size of the Ca cation within the unit cell, and the second type of distortion is due to the Jahn–Teller (JT) effect on the ${Ti}^{3+}$ ions, which distorts the $TiO_{6}$ octahedrons in such a way that there are changes in the Ti-O bond length, and an increase in the angle of the Ti−O−Ti at values close to 180° [56], as observed in the results in Table 3.

#### 3.2.4. Nitrogen Adsorption and Desorption Isotherms

It has been observed that the surface area of inorganic molecules plays an important role in the catalytic activity of some contaminants. High surface areas lead to greater adsorption of the contaminant molecule on the surface of the catalyst and an improvement in its photocatalytic activity [57]. The most common methods to study pores, classifying them according to the IUPAC into micropores (<2 nm), mesopores (2–50 nm), and macropores (>50 nm), are low-temperature nitrogen sorption and mercury porosimetry [58]. The first method is based on nitrogen physisorption, which is the best known, validated, and accepted method for determining the specific surface area of the particles that are part of a solid. This technique uses Brunauer, Emmet, and Teller (BET) physical adsorption (physisorption) isotherms to determine the surface area and pore size distribution of different materials. In this type of isotherm, a layer of inert gas of a monomolecular thickness is formed, and on top of it, multilayers of gas are deposited that interact weakly through forces of the Van der Waals type. Its limitations are due to the fact that nitrogen sorption at 77 K in smaller micropores <0.7 nm occurs at relative pressures of $10^{-6}–10^{-5}$, which requires high precision barometers and significantly longer equilibrium times. Furthermore, some materials are characterized by heterogeneities in the distribution of pores and nitrogen adsorption at low temperature, which does not allow for determining the global porosity, including the heterogeneity of the internal and external layers, affecting the specific area of the material [58]. The second method, called mercury porosimetry, determines the macroporosity (pore size > 50 nm), considering that sorption in the macropores occurs at pressures close to the condensation pressure, which does not allow its volume to be estimated with sufficient precision by means of gas sorption. This method is not applicable in the micro- and mesopore range, due to the limited pressure applied in the system.

**Figure 5:**
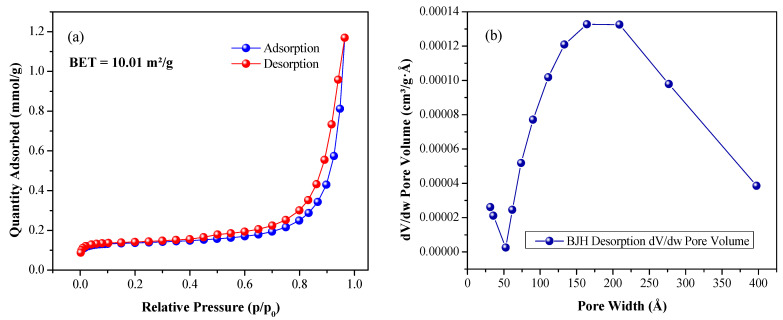
(**a**) Adsorption and desorption isotherms of nitrogen; (**b**) pore size distribution curve.

In this research, the specific surface area, volume, and pore size distribution of the synthesized material were obtained through $N_{2}$ adsorption and the BET method. The corresponding adsorption–desorption isotherms of the CTO NPs are shown in Figure 5a; these can be classified as type IV curves, according to the IUPAC classification [40], and the shape of the hysteresis loop corresponds to the H3 type according to the same classification. This indicates that the CTO synthesized using the Pechini method can be classified as a mesoporous material. The presence of a quantity of micropores is also strongly indicated by the near-vertical adsorption isotherm at the lowest relative pressures analyzed, that is, in the range of 0.014−0.030 (initial left part of Figure 5a). Specifically, the adsorption branch of this isotherm (Figure 5a) showed a gradual increase in the volume of gas absorbed, in the range of low relative pressures, from approximately 0.030 to 0.83, subsequently showing a strong increase from 0.83 to 0.96 for higher relative pressures of the gas, a behavior that would indicate the presence of mesopores. The desorption branch of the isotherm is quite similar for values less than 0.40 (Figure 5a). For higher values, a small hysteresis loop is observed, which would indicate the presence of mesopores.

To determine the surface area, the Brunauer, Emmet, and Teller (BET) method [59] was used, considering that the range of relative pressures where the adsorbate monolayer is formed and where the data fit the linearized form of the BET equation is from 0.05 to 0.25. Thus, the results of the BET surface area measurement show a value of $10.01\;m^{2}/g$ of CTO.

The results of the porosity analysis for the perovskite synthesized using the Pechini method, meanwhile, are indicated in the Barret–Joyner–Halenda (BJH) pore volume curve (Figure 5b); this shows a broad distribution centered at ~185 Å. To calculate the total pore volume and mean pore diameter, the desorption isotherm data were used. A total pore volume of $0.038739\;{\mathrm{cm}}^{3}/g$ and a mean pore diameter of 184.869Å (~18.5nm) were obtained. It is noteworthy that the largest number of pores present in the sample are larger than 5nm in Figure 5b, indicating that the synthesized CTO is a mesoporous material.

### 3.3. Optical Characterizations

#### 3.3.1. Fourier Transform Infrared Spectroscopy

**Figure 6:**
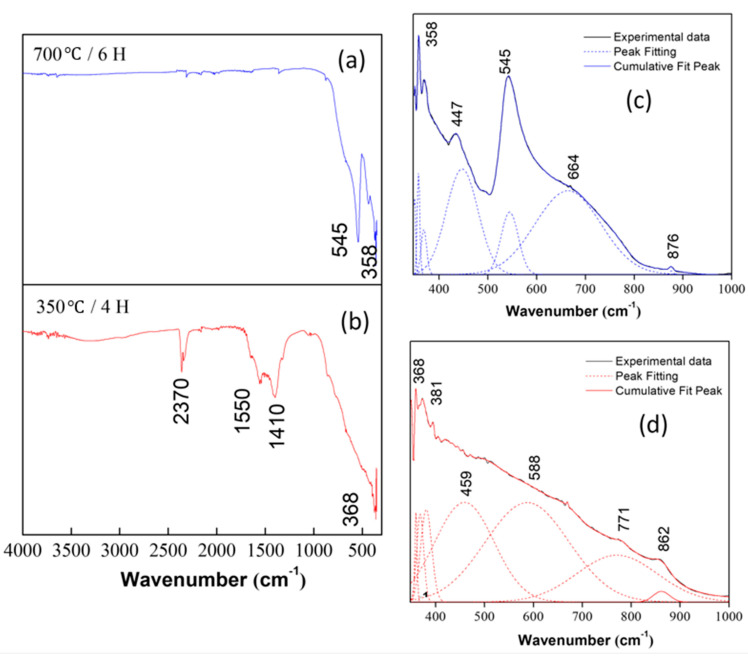
IR spectra of the $CaTiO_{3}$ system heat-treated at (**a**) 700°C and (**b**) 350°C, (**c**,**d**) shows the result of the mathematical process of deconvolution, in the range between 350 and $1000\;{cm}^{-1}$, of the IR spectra in (**a**) and (**b**), respectively, the solid black lines show the FTIR spectra, the blue and red dotted lines indicate the deconvolutions.

In order to analyze the effect of heat treatment on the synthesized ceramic powders of the $CaTiO_{3}$ system obtained using the Pechini method, IR spectra of the samples that were heat-treated at 350 and 700°C were taken. Figure 6 shows three regions, with the first between 4000 and 2000 ${cm}^{-1}$, which can be associated mainly with vibrational modes of the ${OH}^{-}$ hydroxyl groups present in the sample. The second is between 2000 and 1000 ${cm}^{-1}$ and gives information about the vibrational modes of the carbon-containing bonds. The last region, $1000–400\;{cm}^{-1},$ would give information about the vibrational modes of the oxide of interest [49]. In the spectrum of Figure 6a, corresponding to the sample treated at 350°C, at around $3300–3500\;{cm}^{-1}$, the overlapping of the vibration bands of the hydroxyl group and the stretching vibration of the adsorbed water molecule can be observed [60,61]; the vibration corresponding to the N−H bond is observed near $\sim 2300\;{cm}^{-1},$ as well as the vibrational modes due to the symmetrical stretching of the C−H bond between ~1400 y $1550\;{cm}^{-1}$ [62].

The bands below $800\;{cm}^{-1}$ of Figure 6c,d, are mainly associated with functional groups that contain the Ca−Ti−O bonds and that exist in the synthesized compounds. These bands correspond to bonds formed by titanium with other species and are, therefore, the area that provides more information about the nature of the Ca−Ti complexes that exist in the system. 

In addition, in the spectra in Figure 6a,b, it is observed that, when thermally treating the samples, the bands located between 1400 and $2380\;{cm}^{-1}$ decrease. This indicates that the heat treatment was effective in removing water or impurities from the system. If the IR spectra corresponding to the sample at 350°C and the one thermally treated at 700°C are compared, it can be concluded that, in the second spectrum, the formation of the structure corresponding to the $CaTiO_{3}$ system [63] was favored. Figure 6c,d show the deconvolutions of the spectra presented in Figure 6a,b, respectively; the vibrational modes associated with each band can be seen in Table 4.

In Figure 6b, the band around $\sim 381\;{cm}^{-1}$ is assigned as an extension of the Ti−O bond, characteristic of the $TiO_{2}$ vibration mode. This band is sensitive to a change in the angle of this bond, related to the $TiO_{6}$ octahedron of $CaTiO_{3}$ and, therefore, influenced by the $TiO_{2}$ phase shift with increasing temperature [47], the presence of a $TiO_{2}$ phase is also evident in the XRD analysis.

#### 3.3.2. Raman Spectroscopy

**Figure 7:**
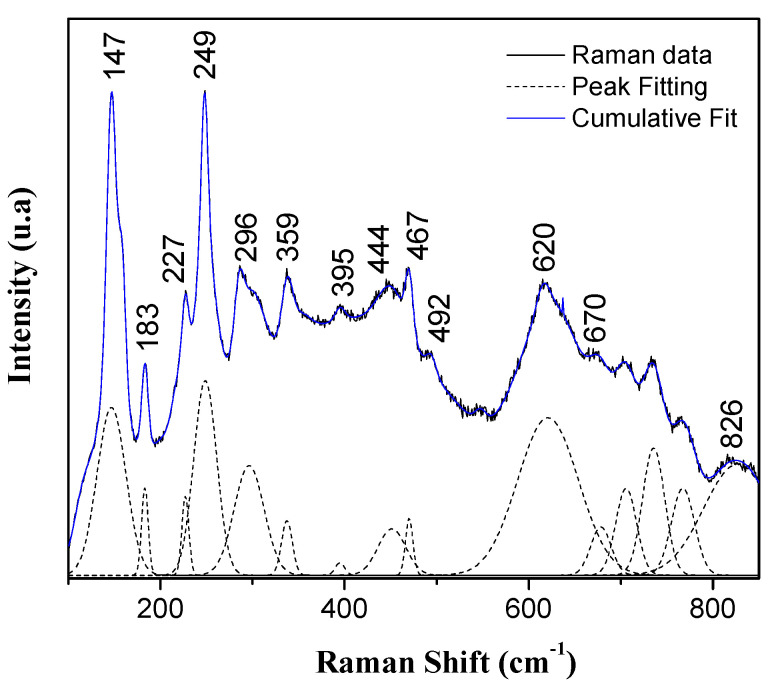
Raman spectrum of the CTO system obtained at 700 °C. The solid black line shows the Raman data, the black dotted line corresponds the fitting result of the mathematical process of deconvolution and solid blue line indicates the cumulative fit.

The Raman spectrum corresponding to the CTO system treated at 700°C is indicated in Figure 7. The spectrum data underwent a deconvolution process using the Fityk 0.9.8 software, which was used to assign the possible active modes in Raman spectroscopy for the studied sample. It has been determined that these systems, with an orthorhombic structure of the Pbnm space group (62), present 60 active Raman modes: twenty-eight associated with dipole moment changes $\left(8B_{1u},{10B}_{2u}\;and\;{10B}_{3u}\right)$, twenty-four symmetric and antisymmetric modes of stretching related to changes in polarizability $\left({7A}_{g},{7B}_{1g},{5B}_{2g},{5B}_{3g}\right)$, and eight modes concerned with cation substitution in site A $\left(8A_{u}\right)$ [69,70,71].

Observing the spectra in Figure 7, a first band located at ~147 ${cm}^{-1}$ can be highlighted, associated with the translation vibration mode between the Ca and $TiO_{3}$ ions within the $CaTiO_{3}$ structure [72]. Subsequently, the bands located at ~183, ~227, ~249, ~296, ~359, ~395, ~467, and ~670 ${cm}^{-1}$ can be attributed to active vibrational modes, $A_{g}$, associated with the bending modes of O−Ti−O, which have been widely reported for the $CaTiO_{3}$ system [69,73,74].

In the spectra, it is possible to observe bands located at ~444−492 ${cm}^{-1}$ that can be attributed to the torsional vibration modes, $B_{1g}$, in the $Ti–O_{6}$ bonds; these modes are related to the internal vibration of the oxygen cage of the structure [73,74]. Finally, the bands at ~620–670 ${cm}^{-1}$ are observed, corresponding to the stretching mode in the Ti−O bond [69] and a band close to 826 ${cm}^{-1}$, associated with the substitution of the calcium ion within the perovskite structure [70]. These results show the effect of the angular deformation of the O−Ti−O bonds, characteristic of the orthorhombic phase observed in the XRD diffractograms of the samples (See Figure 4).

#### 3.3.3. Diffuse Reflectance Spectroscopy

**Figure 8:**
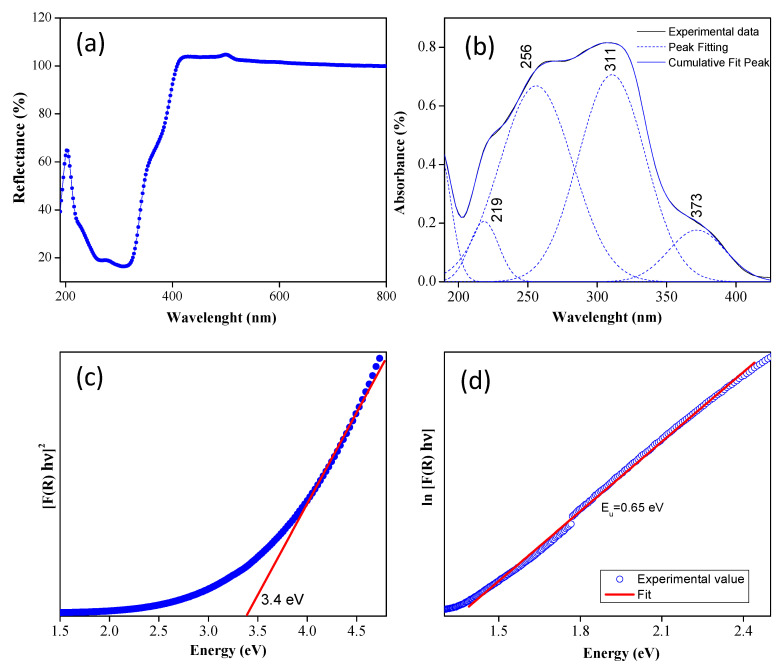
(**a**) DRS UV-Vis spectra corresponding to powders of the $CaTiO_{3}$ systems, (**b**) deconvolution of the absorbance spectrum, (**c**) bandgap energy obtained using the Kubelka–Munk function, and (**d**) determination of Urbach energy ($E_{u}$) from the graph $\ln\left[F\left(R\right)\right]$ vs. hυ.

The $CaTiO_{3}$ system gives a white color, indicating an absorption capacity in the visible light range, a behavior that was evidenced by observing the results of spectroscopy in the UV–visible range that were obtained for this system (Figure 8a). A deconvolution process was carried out to determine the different absorption bands in the UV-Vis spectra of the sample of interest. This system showed UV light absorption mainly at the 256nm and 311 nm wavelengths (Figure 8b). These UV absorption bands are generated by the intermolecular transition process of charge transfer of the π → π∗ orbitals, a result that is consistent with what has been observed in the literature [53,60]. The band at 373nm is due to light absorption caused by the excitation of electrons from the valence band to the conduction band of $CaTiO_{3}$ [75].

The bandgap energy value $\left(E_{g}\right)$ was determined from the Kubelka–Munk function, which can be expressed in the following equation:

(3) $$F\left(R\right)\times h\nu =A{\left(h\nu -E_{g}\right)}^{n}$$

where F(R) is the Kubelka–Munk function, h is Planck’s constant, ν is the frequency of light, A is the absorption constant, and n is the coefficient associated with the electronic transition that can take values of ½ and 2, if the transition is direct or indirect, respectively. In this case, n=1/2 was considered because these particles could present direct transitions; the $E_{g}$ value was calculated from the resulting curve by plotting $\left(F\left(R\right)\times h\upsilon \right)\times R^{2}$ as a function of hυ, extrapolating the linear part towards the abscissa axis.

Figure 8c shows the transformation of the Kubelka–Munk function, the result from which a value of the energy bandgap of 3.4 eV was obtained, considering a direct transition, which is important to avoid an increase in the recombination of the electron–hole pair, which potentiates these materials for their use in photocatalytic activity [61]. When the $E_{g}$ obtained in this work is compared for powders synthesized using the Pechini polymeric precursor method, with respect to the values reported for systems synthesized through the hydrothermal and sol–gel methods, with values of 3.42 and 3.49eV, respectively [53], it is observed that there were no significant changes in the gap energy value.

Finally, the Urbach energy ${(E}_{u}$) was determined, originated by the states located in the bandgap, which is related to the disorder in the structure and is given as $\alpha =\alpha_{o}\;exp\;(h\upsilon \;/\;E_{u})$, where α is the absorption coefficient proportional to F(R), hυ is the photon energy, and $E_{u}$ the Urbach energy. From the results obtained from the Kubelka–Munk function, it is possible to calculate the Urbach energy by plotting ln[F(R)] against hυ. The reciprocal of the slope of the fit of the linear part of this curve, Figure 8d, observed below the optical band gap, will be equal to the value of $E_{u}$. The calculated Urbach energy presented a value of 0.65 eV, a result that indicates a greater disorder in its structure and/or a greater contribution of the defects to the states located in the band gap.

#### 3.3.4. Photoluminescence Spectroscopy

**Figure 9:**
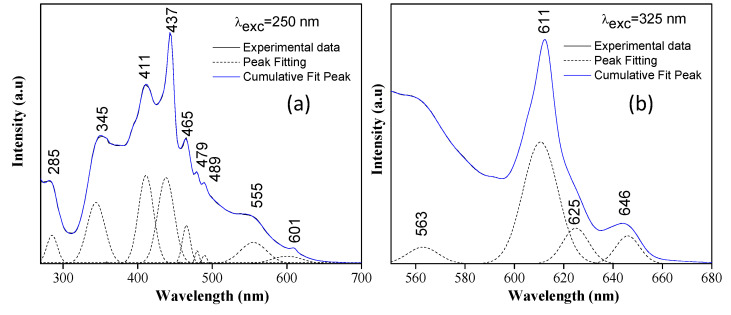
CaTiO_3_ photoluminescence spectra obtained with excitation lengths 250 nm (**a**) and 325 nm (**b**). The solid black line shows the experimental data, the black dotted line corresponds the fitting result of the mathematical process of deconvolution and solid blue line indicates the cumulative fit.

Figure 9 presents the photoluminescence (PL) spectra corresponding to the $CaTiO_{3}$ system, synthesized at 700 °C, using two excitation wavelengths of 250nm (Figure 9a) and 325nm (Figure 9b). The PL emission can change due to the presence of defects such as oxygen vacancies (*V*_O_) and Ti vacancies (*V*_Ti_) that affect the optical properties of these systems. Figure 9 shows that the perovskite exhibits strong luminescence upon ultraviolet excitation.

In the emission spectrum of Figure 9a, a broad band of luminescence is observed in the range of ~280−650nm, showing that these systems present a strong emission in the visible light region. The PL spectrum of Figure 9a was characterized by two bands located at ~411nm (3eV) and ~437nm (2.8eV), both in violet, with the former attributed to emission from the intermediate state directly below the conduction band [76] and the latter to the delocalized electronic levels associated with the transfer of charge between the O(2p)–Ti(3d) bands, due to the effects of the crystalline field of the material [77]. The bands between ~465nm and ~479nm (blue), meanwhile, are characteristic of $CaTiO_{3}$ systems with perovskite structures. These bands were related to the radioactive recombination of the electron–hole pair trapped in the octahedral structural unit of $TiO_{6}$ in the $CaTiO_{3}$, known as the self-trapped exciton (STE) state [76]. In the spectrum, it is possible to observe bands between 500 and 650nm with a lower emission intensity, an effect attributed to the recombination rate of the electron–hole pairs, and which can improve the photocatalytic activity [61]. A band appears in this region at ~601nm, corresponding to orange (2.1 eV), which indicates the presence of ${Ti}^{3+}$ ions located in octahedral sites, related to the strong crystalline field of the structure. Furthermore, these bands suggest the presence of surface defects and oxygen vacancies in the system [60,78]. 

In Figure 9b, the emission spectrum obtained with a 325nm excitation source is shown, the spectrum presents a luminescence band in the range of ~560−680nm, with defined maxima at 611 (orange/2.03 eV) and 646nm (red/1.92 eV). The profile obtained may be due to the participation of numerous states within the bandgap of the material, caused by intrinsic defects in the material [44,73]. These defects are directly related to the oxygen vacancies $\left(V_{O}^{∙}\right)$ in complex groups or in the torsion of the bonds between the $TiO_{6}-TiO_{6}$ groups [79]. The presence of titanium ions, and the structural defects of these systems, together with the value of $E_{g}$ obtained from the diffuse reflectance spectroscopy tests (Figure 8c), suggest that the studied system presents a semiconductor behavior with potential applications in photocatalysis [44] and optical devices [77]. Table 5 summarizes the possible defects associated with the bands of the PL spectra.

### 3.4. Methyl Orange (MO) and Levofloxacin (LVF) Photodegradation Tests

#### 3.4.1. Determination of Calibration Curves

To evaluate the degradation capacity of MO and LVF by the $CaTiO_{3}$ compounds, the UV-Vis spectroscopy technique was used. Initially, the relationship between the absorbance recorded with the spectrophotometer and the concentration of each contaminant was determined, obtaining the respective calibration curves. UV-Vis absorption spectra were obtained using a Jasco V700 UV-VIS spectrophotometer, in the range of 200–600 nm.

**Figure 10:**
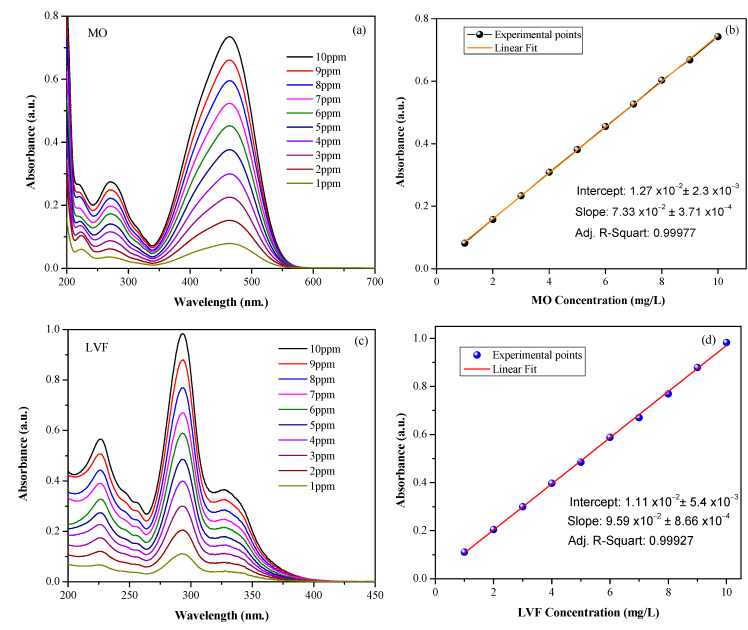
UV-Vis absorption spectra corresponding to solutions of (**a**) MO and (**c**) LVF in concentrations between 1 and 10 ppm. Calibration curves of (**b**) MO and (**d**) LVF to determine the amount of contaminant present in a solution using the maximum peak absorbance value (~464nm for MO and ~293nm for LVF).

Initially, a stock solution of MO was prepared at 1000 ppm (mg/L) in deionized water. From this, a 10 ppm dilution was obtained and its UV/Vis spectrum was determined (see Figure 10a), in which the intensity (absorbance value) corresponding to the maximum absorption peak at a wavelength value of ~464 nm was recorded, a parameter that was chosen to determine the concentration of MO in aqueous solutions. MO solutions between 1 ppm and 10 ppm (Figure 10a) were made in order to determine the UV/Vis spectroscopy calibration curve, which allowed for the measuring of the absorbance as a function of concentration, as indicated in Figure 10b. It can be seen that the fit of the curve is maintained with an adequate linear regression parameter ($R^{2}=0.99977$).

A stock solution of LVF was prepared in a similar way at 2000 ppm (mg/L) in demineralized water. From this, a 10 ppm dilution was obtained and its UV/Vis spectrum was determined (Figure 10c). In the LVF spectrum, two absorption peaks were identified in the ultraviolet region with maximums at ~226 nm and 293 nm. The second one presents a much higher relative intensity, which is why it was decided to work at this wavelength to determine the LVF concentration in aqueous solutions. LVF solutions were made between 1 ppm and 10 ppm (Figure 10c) and a calibration curve was obtained (Figure 10d) with an appropriate linear regression parameter ($R^{2}=0.99927$).

#### 3.4.2. MO and LVF Degradation Kinetics

**Figure 11:**
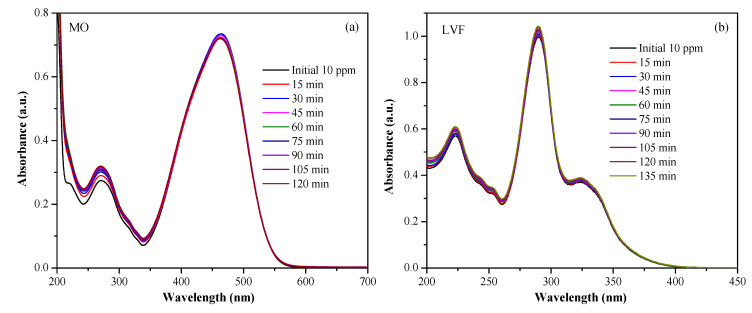
Photolysis of MO (**a**) and LVF (**b**).

The photocatalytic activity of the CTO was investigated using MO and LVF as target molecules. Initially, the pollutant degradation test was performed in the absence of a photocatalyst to confirm whether the degradation of MO and LVF was photocatalytic or photolytic in nature. Solutions of 50 mL at 10 ppm of each of the contaminants were prepared, and they were placed in a photoreactor equipped with a UV lamp; aliquots of the solutions were taken at 15 min intervals and the absorbance spectra were measured. It was observed that MO and LVF are stable under UV radiation; Figure 11a, b shows the photolysis of MO and LVF, respectively, Figure 11 indicates that there was no representative change in the aqueous solution with the contaminants exposed to radiation in either case. The UV-Vis spectra of the aliquots of the solutions taken at different times were similar, which means that the degradation of MO and LVF due to the effect of radiation can be considered insignificant. 

**Figure 12:**
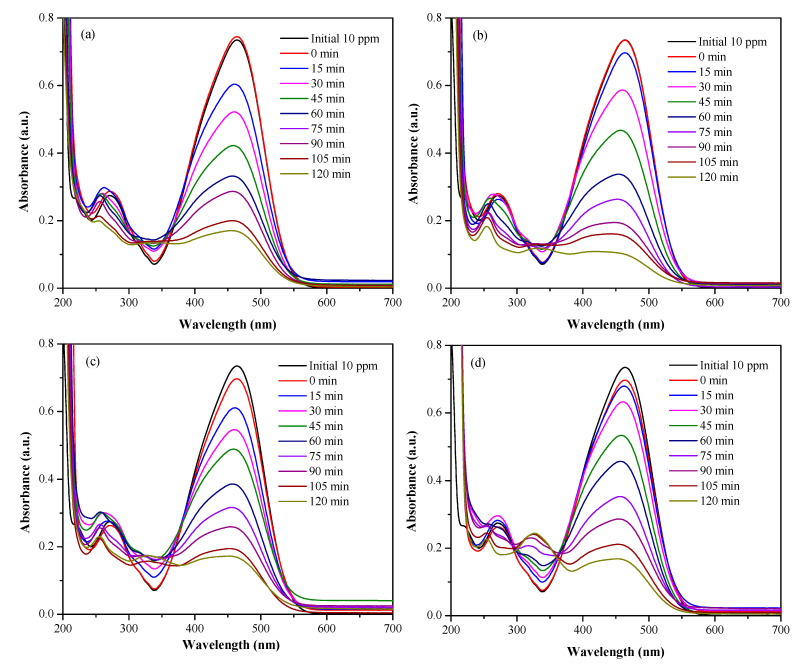
UV-Vis absorption spectra corresponding to solutions of MO and CaTiO_3_ nanoparticles in concentrations of (**a**) 3 g/L, (**b**) 5 g/L, (**c**) 10 g/L, and (**d**) 15 g/L.

**Figure 13:**
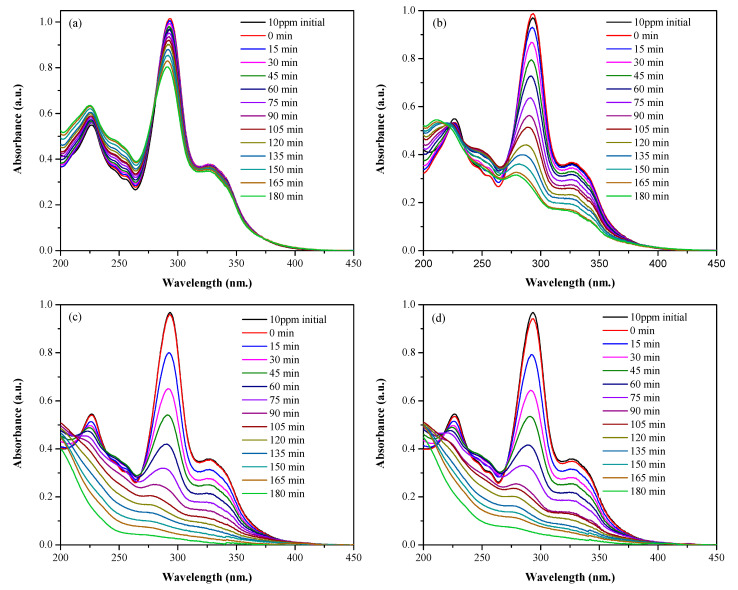
UV-Vis absorption spectra corresponding to LVF solutions and CaTiO_3_ nanoparticles at concentrations of (**a**) 3 g/L, (**b**) 5 g/L, (**c**) 10 g/L, and (**d**) 15 g/L.

The graphs of the degradation of MO and LVF, using the CaTiO_3_ nanoparticles at different concentrations, are presented in Figure 12 and Figure 13, respectively, Figure 12a–d and Figure 13a–d present the degradation of the contaminants for catalyst doses of 3,5,10 and 15 g/L, respectively. Initially, the CTO NPs are brought into contact with the contaminants under constant stirring for 30 min and in the absence of radiation to establish the adsorption–desorption equilibrium. After this time, an aliquot of the solution is taken, the NPs are removed via centrifugation, and the absorption spectrum is measured. These measurements are labeled as 0 min in Figure 12 and Figure 13. Once the adsorption–desorption equilibrium is established, the UV lamp is switched on. The results show a decrease in the absorption maximum as the time of exposure to UV radiation increases, which indicates the degradation of the pollutants. The kinetic mechanism most associated with photocatalysts with a composition similar to that of this work, acting on organic compounds, is the first-order linear mechanism [16,80,81,82]. This model, also known as the “Langmuir−Hinshelwood mechanism” describes photocatalytic degradation, including the sorption effect. In this model, the reagents are adsorbed on the active sites before the start of the reaction [83]. The expression that explains the kinetics of the catalytic processes is given through the pseudo-first-order equation (Equation (4)):

(4) $$ln\left(\frac{C_{o}}{C_{f}}\right)=kt$$

where k is the kinetic degradation constant, also called the apparent rate constant (Kapp, min^−1^), *C_o_* and *C_f_* are the initial concentration of the pollutant (mg/L) at time 0 and during irradiation at time *t*, respectively, and *t* is the time (min) [81,83].

**Figure 14:**
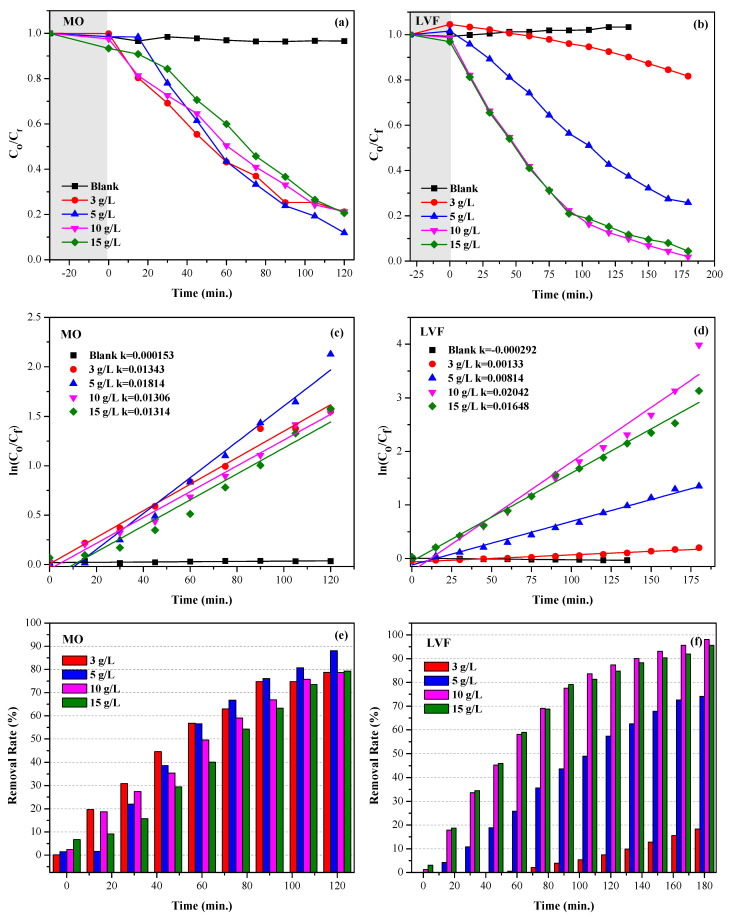
(**a**,**b**) Photocatalytic activity of CTO with MO and LVF for different catalyst concentrations. (**c**,**d**) Graphs of photocatalytic degradation kinetics and determination of reaction rate constant. (**e**,**f**) Percentages of degradation as a function of time.

Figure 14a–d show the kinetic study, using the nanoparticles of the CTO system. The value of the apparent velocity constants (K) is calculated from the slope of the graph of $ln\left(\frac{C_{o}}{C_{f}}\right)\;$ versus time, using the expression in Equation (4). The kinetic parameters for the degradation of MO and LVF, the K values, and the calculated fit factors are shown in Figure 14c,d and in Table 6. The results revealed that the MO and LVF photodegradation process is consistent with the pseudo-first-order reaction kinetics model. 

Measurements made at 0 min (Figure 12 and Figure 13) indicate that, in the absence of light, the CTO samples do not exhibit contaminant adsorption. However, upon irradiation with ultraviolet light in the presence of the nanoparticles, the contaminants undergo a marked degradation. Figure 14c,d show that for MO and LVF, the maximum CTO reaction rate constants are $0.01814\;{min}^{-1}(dosis\;5\;gL^{-1})$ and $0.02042\;{min}^{-1}(dosis\;10\;gL^{-1})$, respectively. After 2 h of treatment with CTO and UV irradiation (Figure 14c, Table 6), the dye degradation rate reached 78.8, 88.1, 78.7, and 79.4%, for concentrations of 3, 5, 10, and 15 $gL^{-1}$, respectively. When the catalyst dose is increased from 3 to 5 $gL^{-1}$, the active sites increase; therefore, the percentage of degradation increases. If the dose continues to increase, the degradation efficiency decreases. This may be due to the fact that the excess of catalyst in the solution hinders the transmission of light.

Similarly, in Figure 14c and Table 6, we observe that the degradation rates of the antibiotic after 3 h of irradiation reach 18.3,74.2,98.1,and95.6% for concentrations of 3, 5, 10, and 15 $gL^{-1}$, respectively. If the dose of the catalyst is increased from 3 to 10 $gL^{-1}$, the percentage of LVF degradation increases, but if the CTO dose is raised to 15 $gL^{-1}$, the degradation efficiency decreases. This implies that 5 $gL^{-1}$ and 10 $gL^{-1}$ are the optimal catalyst doses for the MO and LVF degradation systems, respectively.

To explain the results of the MO and LVF degradation by the CTO-NPs, it is pertinent to initially consider the adsorption process of MO and LVF molecules on the surface of the NPs. Despite the small particle size of these solids (Figure 3), and the specific area reported in the BET analyses, the adsorption process was not very important (Figure 12 and Figure 13); however, photocatalytic activity was found to occur.

**Figure 15:**
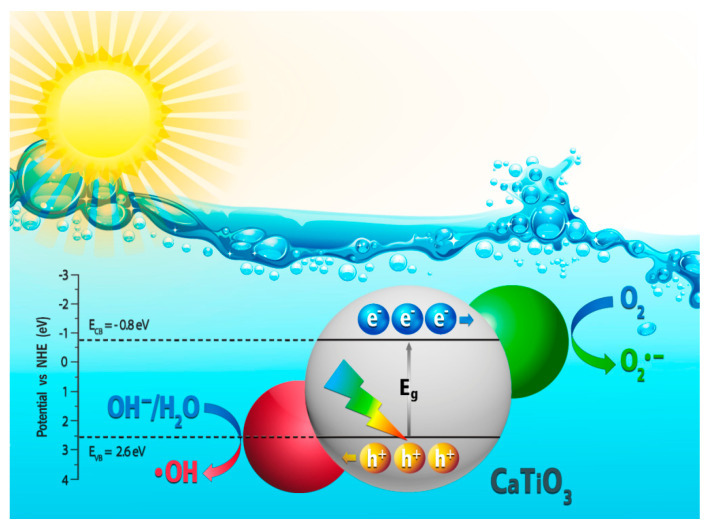
Schematic illustration of the photocatalytic mechanism of the CTO NPs.

To account for the possible fundamental mechanisms of photocatalysis of CTO, we ought to bear in mind that the synthesized material is a semiconductor that shows optical absorption in the UV region of the spectrum (λ<400nm), with a bandgap energy value of $E_{g}=3.4\;eV$ (Figure 8). Figure 15 schematically shows the photocatalytic mechanism of the CTO in the degradation of MO. When the CTO is photoinduced using sunlight with a photon energy (hν) equal to or greater than the bandgap energy ($E_{g}$), the electrons in the valence band (VB) are promoted to the conduction band (CB). Therefore, highly active electrons $\left(e^{-}\right)$ are generated in the CB, and positively charged holes (h^+^) are formed in the VB [46]. Simultaneously, a fraction of the photogenerated $e^{-}$ and h^+^ recombines. Only those that survive recombination reach the semiconductor surface and interact with the dissolved oxygen to form the superoxide radical anion ($•O_{2}^{-}$). The conduction band potential of the CTO is calculated to be −0.8eV vs. a normal hydrogen electrode (NHE) using the following relation [84]:

(5) $$E_{CB}=X-E^{e}-0.5E_{g}$$

where X is the absolute electronegativity of the CTO (estimated to be 5.4eV, according to the data reported in the literature [84]), $E^{e}$ is the energy of free electrons on the hydrogen scale (4.5eV), and $E_{g}$ is the bandgap energy of the CTO (3.4eV) [84].

Moreover, the water molecules adsorbed on the surface of the CTO interact with $h^{+}$ to form hydroxyl radicals (•OH) [31]. The CB background of CTO (−0.8eV) is more negative than the standard redox potential of $O_{2}/•O_{2}^{-}$ (−0.33 eV), indicating that these excited electrons in the CB of CTO can react with adsorbed oxygen and generate $•O_{2}^{-}$ [10]. Furthermore, the potential of the VB of the CTO is more positive than that of the H_2_O/•OH (1.99 eV), so the $h^{+}$ in the VB of the CTO can react with $H_{2}O$ to generate •OH [10]. The $•O_{2}^{-}$ and •OH radicals have strong oxidizing properties and can decompose organic pollutants into other intermediates or small molecules such as $CO_{2}$, $H_{2}O,$ and mineral acids [16,46]. The degradation processes are the following (Equations (6)–(10)):

(6) $${CaTiO}_{3}+h\nu \to {CaTiO}_{3}(e_{CB}^{-})+{CaTiO}_{3}(h_{VB}^{+})$$

(7) $${CaTiO}_{3}(h_{VB}^{+})+H_{2}O\to h^{+}+•OH$$

(8) $${CaTiO}_{3}(e_{CB}^{-})+O_{2}\to •O_{2}^{-}$$

(9) $$Pollutants+•OH+•O_{2}^{-}\to intermediates$$

(10) $$Intermediates\to Degradationproducts+{CO}_{2}+H_{2}O$$

For comparison, Table 7 presents the photocatalytic performances of the CTO nanoparticles used in the degradation of some pollutants that have been reported in the literature. The results obtained with the particles synthesized using the polymeric precursor method reported in this work had a lower efficiency of degradation of MO, with a degradation percentage of 88.1% at a concentration of 5g/L and a time of 120 min, compared to other chemical methods, such as the thermal method with an efficiency of 99.03% at a concentration of 0.3g/L and a time of 40 min [85]. Generally, several parameters inhibit degradation, including the amount of the catalyst, the reaction temperature, the pH, the nature and concentration of contaminants, and the nature of inorganic ions [85]. As shown in Table 7, the amount of the CTO photocatalyst used to degrade MO in the present work was greater than those reported for similar systems, which would affect the speed of the photocatalytic reaction; that is, when the amount of the photocatalyst is greater than the saturation point, the light photon adsorption coefficient decreases and, consequently, the photocatalytic efficiency decreases, inhibiting degradation [44]. Moreover, it has been reported that the morphology of the nanoparticles affects the photocatalytic performance and efficiency of the degradation of the MO dye. As could be seen in the SEM images in Figure 3, in the present study, granulated nanoparticles with an irregular morphology were obtained at a lower efficiency in the degradation of MO, while those obtained using the thermal method for the same system [86], with a rod-like morphology, achieved double the photocurrent density versus granulated CTO particles, resulting in a greater inhibition of the charge carrier recombination and greater photocatalytic activity. This effect may be related to the increase in the surface area of the particles, which was $10.01\;m^{2}/g$ in this work, compared to higher values of CTO systems, obtained through the hydrothermal method, with a surface area of $18.20\;m^{2}/g$, at an efficiency of 96.6% in 60 min, by dispersing 0.1 g of CTO in 200 mL of an aqueous solution of 10 mg/L of MB. It has been proposed that a higher surface area value promotes the generation of active species (•OH, $•O_{2}^{-}$, $h^{+}$) to decompose contaminants [53]. 

Another factor to consider is the variation in pH, which can influence the adsorption of the contaminant molecule on the surface of the CTO. Acidic solutions (pH < 5) inhibit degradation due to the high concentration of protons. Studies show that the removal rate of methyl blue increases with increasing solution pH, given that the MO contaminant is a positively charged cationic system, which is more easily attracted to the surface of the material through the electrostatic interaction in alkaline conditions, favoring its degradation. That is, under neutral and alkaline conditions, it can be removed up to almost 100% [16]. This same study revealed that by synthesizing a $CaTiO_{3}/g-C_{3}N_{4}$ heterojunction with 40% $C_{3}N_{4}$, 87.7% of LVF can be removed in a time of 120 min with a concentration of 20 mg/L at pH 7. This behavior is due to the fact that LVF molecules behave like zwitterions at pH values of 6.02−8.15, which facilitates the reaction of $h^{+}$ in the VB of the CTO with $H_{2}O$ to generate •OH, $•O_{2}^{-}$ and •OH, these active species have strong oxidant properties and can decompose contaminants such as LVF, mainly through the oxidation of the piperazine ring, that finally decomposes into amines and carboxylic acids, which are small compounds that, over time, could mineralize into small molecules. The opposite happens at pH 9–11, since LVF molecules behave like anions, repelling electronegative material, resulting in lower removal rates; that is, the alkaline state exerts an obvious inhibitory effect on LVF degradation [16,87].

Therefore, it is necessary to make adjustments to the synthesis conditions of the CTO nanoparticles, which allow for the control of the morphology of the particles and an increase in the surface area. Some alternatives to be considered may be the doping or composition of heterostructures with some metallic elements, including Zr [88], Fe [89], Ag [65], graphene [84], and g-C3N4 [90], which have shown an increase in photocatalytic activity. These results suggest possible alternatives for producing CTO with a higher percentage of degradation of emerging contaminants such as LVF in the future.

**Table 7:** Some methods of synthesis for obtaining the CaTiO_3_ system.

System	Method of Synthesis	Parameter of Synthesis	$E_{g}$ (eV)	$\mathrm{Surface}\;\mathrm{Area}\;(m^{2}/g)$	Emerging Pollutant	Irradiation	Efficiency (%)	Ref
$CaTiO_{3}$	Sol–gel	Precursor: Calcium chloride (CaCl_2_), Titanium (IV) isopropoxide [Ti(OC_4_H_9_)_4_] 700 °C/2 h	2.95	48.2	MO	100 min Light source: 500 W Xe lamp (470 nm).	62% 10 mg/50 mL of 5 mg/L	[60]
		900/2 h	2.82	73.4	MO		88% 10 mg/50 mL of 5 mg/L	
$CaTiO_{3}$	Microwave-assisted method	Precursor: Calcium acetate Ca(CH_3_COO)_2_2H_2_O, Titanyl sulfate TiO(SO_4_.) Microwave irradiation at 400 W	2.67	-	MB	240 min Light source: 6 W Hg lamp (254 nm)	96.4% 0.1 g in 20 ppm de MB dye	[44]
$CaTiO_{3}$	Polyacrylamide gel route	Precursor: calcium nitrate Ca(NO_3_)_2_4H_2_O, Titanium Tetrabutoxide Ti(C_4_H_9_O)_4_Chelating agent: Ethylenediaminetetraacetic acid (EDTA) 600 °C/6 h	3.66	60.5	MO	180 min Light source: 15 W low-pressure mercury lamp (254 nm)	96% 0.1 g/100 mL of 1 mg/L	[91]
$CaTiO_{3}$	Solid state	Precursor: calcium carbonate CaCO_3_, titanium oxide TiO_2_ 1400 °C/3 h	3.18	0.10	MB	60 min Light source: 500 W Hg lamp	0.1 g/200 mL of 10 mg/mL	[53]
	Sol–gel	Precursor: calcium nitrate tetrahydrate Ca(NO_3_)_2_·4H_2_O, Tetrabutyl titanate Ti(C_4_H_9_O)_4_ 700 °C/3 h	3.42	9.69	MB		0.1 g/200 mL of 10 mg/mL	
	Hydrothermal	Precursor: calcium nitrate tetrahydrate Ca(NO_3_)_2_·4H_2_O, Tetrabutyl titanate Ti(C_4_H_9_O)_4_ 200 °C/24 h.	3.49	18.20	MB		96.6% 0.1 g/200 mL of 10 mg/mL	
$CaTiO_{3}$	Thermal method	Precursor: calcium nitrate Ca(NO_3_), Tetra-n-butyl titanate C_16_H_36_O_4_Ti 700 °C/5 h	3.08	-	MO	40 min Light source: 500 W, Xe lamp	99.03% 15 mg/50 mL of 10 mg/L (0.3 g/L)	[86]
$CaTiO_{3}$	Hydrothermal	180 °C/12 h Precursor: titanium isopropoxide	3.65	108.14	Arsenite [As(III)]	40 min Light source: UV light irradiation (254 nm)	98.4% 80 mg/80 mL of 2 mg/L	[92]
$CaTiO_{3}/gC_{3}N_{4}$	Hydrothermal	180 °C/24 h Precursor: tetrabutyltitanate (TNB), calcium nitrate and melamine. Composition: CTO/40%CN	3.35	-	LVF	120 min Light source: 500 W low-pressure mercury lamp (254 nm)	87.7% 0.4 g/L	[93]
$CaTiO_{3}$	Polymeric precursor method	Precursor: calcium acetate [Ca(C_2_H_3_O_2_)_2_], titanium butoxide (C_16_H_36_O_4_Ti) 700 °C/2 h	3.44	34.6	MB	180 min Light source: 125 W mercury lamp	69% 1.0 g/L and pH of 11.2	[43]
$CaTiO_{3}$	Polymeric precursor method	Precursor: titanium tetrabutoxide $Ti[O{\left(CH_{2}\right)}_{3}CH_{3}$]_4_, and calcium acetate ($Ca{\left(CH_{3}COO\right)}_{2}$700 °C/6 h	3.4	10.01	MO	120 min Light source: UV lamp	88.1% 5 g/L and pH of 6.2	This work
					LVF	120 min Light source: UV lamp	98.1% 10 g/L and pH of 4.5	

## 4. Conclusions

In this work, CTO-NPs were successfully synthesized using the Pechini chemical method at 700 °C with a reproducible, controlled methodology. From the XRD results carried out on the catalyst, the coexistence of two crystalline phases is concluded: a majority perovskite-type phase with a Pbnm orthorhombic structure and a minority rutile-type phase of $TiO_{2}$ with a P42/mnm structure, with a crystallite size of ~84 nm. The Raman spectra revealed the effect of the distortions in the lattice associated with defects in the structure such as oxygen vacancies, the twisting of the bonds between the TiO_6_-TiO_6_ groups, or the presence of ${Ti}^{3+}$ ions located in octahedral sites, defects that were also observed in the PL spectrum. These particles presented a mesoporous structure, with a surface area of $10.01\;m^{2}/g$ and a pore size of ~18.5nm, where an irregular morphology predominated, as well as a high degree of agglomeration. From the UV-Vis DRS analyses, an energy gap of 3.4eV was obtained for the CTO, characteristic of semiconductor materials. Regarding the evaluation of the photodegradation capacity of the nanoparticles obtained on the CEs studied, MO and LVF, with continuous UV radiation, it was found that ~88.1% of the MO degraded with a CTO concentration of 5 g/L (for 2 h of treatment) and ~98.1% of LVF degraded with 10 g/L of CTO (for 3 h of treatment). The kinetics of the reactions were determined to be well represented by pseudo-first-order kinetics. This high photocatalytic performance was attributed to the intrinsic characteristics of CTO, its surface area, and the presence of defects in the structure, which could be active sites or modify the electronic structure, either directly or indirectly increasing its photocatalytic characteristics. This work contributes to the development of green and efficient technologies applied to the area of environmental remediation, particularly to bringing forward friendly, efficient catalysts for the degradation of emerging contaminants, a topic of great current interest.

## Figures and Tables

**Table 1:** Representative groups and compounds of EPs [4,8].

Groups	Representative Compounds
Flame retardants	Tris (chloroisopropyl) phosphate, tris(2-chloroethyl) phosphate.
Personal care products and cosmetics	Soaps, detergents, disinfectants, humectants, shampoo, conditioner, sunscreen, biocides. bisphenols, triclosan, parabens.
Pharmaceuticals	Antibiotics, anti-inflammatory, psychiatric drugs, antiepileptics, analgesics, estrogens, β-blockers, steroids, veterinary medicine.
Pesticides	Organochlorines, organophosphates, fungicides (triazoles), herbicides, insecticides (neonicotinoids), bactericides, rodenticides, nematicides.
Lifestyle products	Caffeine, nicotine.
Illicit drugs	Amphetamine, cocaine, methamphetamine, heroin, morphine.
Industrial substances	Surfactants, perfluorinated compounds, alkylphenols, dyes, nanoparticles.
Biological agents	Pathogenic bacteria, multi-drug-resistant microbes, antifungal resistance, virus, antibiotic resistance genes.
Unintentional persistent organic pollutants	Dioxins, brominated dioxins, halogenated polycyclic aromatic hydrocarbon, polychlorinated naphthalenes, environmentally persistent free radicals.
Other substances/issues	Rare earth elements, metalloids (selenium), microplastics, radionuclides, food packaging, containers, corrosion inhibitors.

**Table 2:** Toxicological effects of emerging contaminants on humans and biota [10,11,12].

Class of Emerging Contaminant	Toxicity Effect
Pharmaceutical products	In mollusks, they cause genotoxicity, neurotoxicity, and oxidative stress; reduced growth in algae and fish; extensive DNA damage in fish, kidney injury and gill alterations; cytoplasmic and nuclear deformities and alterations in hormonal damage in mammals, including humans.
Personal care and hormonal products	Infertility, hormone-dependent tumors, folliculogenesis, spermatogenesis, steroidogenesis, and breast cancer occur in humans. The production of vitellogenin is induced in rainbow trout fry; reduction in plasma testosterone in goldfish. They are inhibitors of algae growth. Damage to the reproductive system, reduced growth, poor heart function, mortality, immobilization, and oxidative stress have been observed in crustaceans.
Pesticides	There have been harmful effects on the gills of fish; the feminization of male aquatic organisms; in humans, the reproductive and sexual systems are severely affected.
Microplastics	Oxidative stress is generated in sea cucumbers; blockage of the alimentary canal in fish, crabs, mussels, oysters, whales and plankton; in humans, they cause cytotoxicity and reproductive damage.
Flame retardants	Inhibition of growth and reproduction and decreased survival rate of crustaceans and zebrafish.
Organohalides	Male fertility, obesity, and puberty in humans.
Polyfluoroalkyl substances (PFAS)	Dysregulation of thyroid hormones and adverse kidney health in humans.

**Table 3:** Main parameters of the $CaTiO_{3}$ system, obtained via XRD at room temperature.

Composition	90%	10%
Structure	Orthorhombic	Rutile
Spatial group	Pbnm	P42/mnm
a(Å)	5.38	4.59
b(Å)	5.44	4.59
c(Å)	7.64	2.96
α=β=γ	90°	90°
$\mathrm{Volume}\;\left({Å}^{3}\right)$	223.6	62.42
Tolerance factor (t)	0.903	---
Bond length (Ti−O)	1.95	---
Angle (Ti−O−Ti)	157.4	---
Crystallite size (nm)	83.8	
$R_{wp\;}(\%)$	6.93	
$R_{p\;}(\%)$	5.43	
$\chi^{2}$	1.482	

**Table 4:** Main vibrational modes and functional groups of $CaTiO_{3}$.

Sample	$\mathrm{Wavenumber}\;({cm}^{-1})$	Vibrational Mode and Functional Groups	Reference
$CaTiO_{3}$ thermally treated at 350 °C	368	-	
	~381	CharacteristicoftheTi–O bond of the $TiO_{2}$	[47]
	459	Characteristic vibration of alkali titanates	[64]
	~588	StretchingofO−Ti−O bond	[51]
	771	-	
	862	SymmetricalstretchingoftheCa−O−Ca bond	[44]
	~1410	DeformationintheO−H groups plane	[65]
	~1550	$\mathrm{Bending}/\mathrm{stretching}\;\mathrm{of}\;\mathrm{the}\;CO_{3}^{2-}$	[44]
	2370	Carbonate-type species related to the oxide.	[66]
	~3300–3500	Stretching of the O−H bond	[60,61]
$CaTiO_{3}$ thermally treated at 700 °C	358	-	
	369	-	
	~447	StretchingofTi−O−Ti	[67]
	545	StretchingofO−Ti−O	[51]
	664	CharacteristicoftheCa−Ti−O group	[68]
	876	SymmetricalstretchingoftheCa−O−Ca bond	[63]

**Table 5:** Possible defects present in the CTO sample.

λ_exit_ (nm)	λ (nm)	Energy (eV)	Color	Possible Defects	Ref.
**250**	411	3.02	Violet	Emission from the intermediate state directly below the conduction band	[76]
	437	2.84	Violet	Charge transfer between bands O(2p)−Ti(3d)	[77]
	465	2.67	Blue	Radioactive recombination of the electron–hole pair trapped in the TiO_6_ octahedral structural unit in CaTiO_3_	[76]
	479	2.59	Blue		
	601	2.06	Orange	Presence of ${Ti}^{3+}$ ions located in octahedral sis	[60,78]
**325**	563	2.20	Yellow	Defects are directly related to the $\left(V_{O}^{∙}\right)$ oxygen vacancies in complex groups or in the torsion of the bonds between the $TiO_{6}-TiO_{6}$ groups	[79]
	611	2.03	Orange		
	625	1.98	Orange		
	646	1.92	Red		

**Table 6:** Parameters obtained from the fit to a first-order kinetic equation and the percentage of degradation of MO and LVF.

Quantity of CTO	Rate of Degradation of MO in 120 min (%)	Apparent Velocity Constants, MO			Rate of Degradation of LVF in 180 min (%)	Apparent Velocity Constants, LVF		
		K (min^−1^)	R^2^	Standard Error		K (min^−1^)	R^2^	Standard Error
3 gL^−1^	78.8	0.01343	0.98357	6.13047 × 10^4^	18.3	0.00133	0.95909	7.91415 × 10^−5^
5 gL^−1^	88.1	0.01814	0.97314	0.00106	74.2	0.00814	0.98680	2.71512 × 10^−4^
10 gL^−1^	78.7	0.01306	0.98357	5.96089 × 10^−4^	98.1	0.02042	0.96806	0.00107
15 gL^−1^	79.4	0.01314	0.94666	0.0011	95.6	0.01648	0.98944	4.9135 × 10^−4^

## Data Availability

Data are contained within the article.
